# Evaluation of Mechanical Tomato Harvesting Using Wireless Sensors

**DOI:** 10.3390/s101211126

**Published:** 2010-12-07

**Authors:** Silvia Arazuri, Ignacio Arana, Carmen Jaren

**Affiliations:** Department of Projects and Rural Engineering, Universidad Pública de Navarra, Campus Arosadia, 31006 Pamplona, Navarra, Spain; E-Mails: ignacio.arana@unavarra.es (I.A.); cjaren@unavarra.es (C.J.)

**Keywords:** IS-100, electronic fruits, harvest, quality, mechanization, physical damages

## Abstract

The harvesting of processing tomatoes is fully mechanised and it is well known that during harvest, fruits are subjected to mechanical stress causing physical injuries, including skin punctures, pulp and cell rupture. Some wireless sensors have been used for research during recent years with the main purpose of reducing the quality loss of tomato fruits by diminishing the number and intensity of impacts. In this study the IRD (impact recorder device) sensor was used to evaluate several tomato harvesters. The specific objectives were to evaluate the impacts during mechanical harvest using a wireless sensor, to determine the critical points at which damage occurs, and to assess the damage levels. Samples were taken to determine the influence of mechanical harvest on texture, or on other quality characteristics including percentage of damages. From the obtained data it has been possible to identify the critical points where the damages were produced for each one of the five harvester models examined. The highest risk of damage was in zone 1 of the combine—from the cutting system to the colour selector—because the impacts were of higher intensity and hit less absorbing surfaces than in zone 2—from colour selector to discharge. The shaker and exit from the shaker are two of the harvester elements that registered the highest intensity impacts. By adjusting, in a specific way each harvester model, using the results from this research, it has been possible to reduce the tomato damage percentage from 20 to 29% to less than 10%.

## 1. Introduction

Tomato is one of the most important crops in the world with a production of 141 million T in 2009 [1]. It can be divided into two different groups: tomatoes for fresh consumption, 75%, and tomatoes to be processed by the canning industry, 25% [2].

Nowadays, the processing-tomato harvest is totally mechanised and it is well known that during harvest fruits are subjected to mechanical stress causing physical injuries, including skin punctures, pulp breaking and slight reduction of firmness [3,4].

Geyer *et al.* [5] observed that larger fruits have lower firmness, causing an increase in the level of damage. Damage susceptibility was studied by Fenyvesi *et al.* [6]. In their report, they determined this susceptibility by dropping tomatoes from different heights and verifying how the firmness decreased using a non-destructive hand penetrometer, the MGA-1091 (FVMMI Gödöllö). These tests determined the resistance of the tomato to cracking (damage in the skin) and to breakage (damages that affect the pulp).

Van Zeebroeck *et al.* [7] demonstrated, using a discrete element method (DEM), that the radius of curvature influences the risk of bruising because tomatoes with a small radius of curvature absorb more energy compared to those with a larger radius of curvature (at location of impact), this effect is better observed at low impacts (15 N).

During harvesting and unloading of the tomatoes, damage is caused by numerous dynamic loads, impacts and falls in the transfer points and by static loads due to increase of the weight in the transport containers. The main consequence is a loss of product quality. During the 1970s, several sensors including “electronic fruits” were developed with various shapes and different systems of data acquisition and information transference. Many of these electronic instruments were developed to monitor quality in packing lines in order to identify and reduce the number and intensity of impacts [8]. Herold *et al.* [9] used a PMS-60 sensor to reduce damages. They observed that during harvesting the load peaks on the tomato were about 60 N, although sometimes they reached 100 N. The largest force peak registered, over 200 N, occurred during unloading onto an empty truck. These impacts caused damage to the tomato; the tomato samples before transport showed damage levels of 43%. After transport the damage to these fruits had increased to 66%. This very high degree of damage does not seem to be typical in a normal harvest [9]. However, if a real degree of visible and invisible damage is assumed, then considerable additional fruit damage could be expected because of the numerous mechanical loads during transport and not related to maturity, variety and other factors [6].

Through years of research sensor or electronic instruments have been used in many approaches with one main purpose: to reduce the quality loss of tomato fruits by diminishing the number and intensity of impacts in chains of mechanised crop production [10].

The most common and abundantly described types are the PMS-60, a pressure measuring sphere, made at the Institute for Agricultural Engineering of Bornim, Germany [11] and Techmark’s IS-100 (MI, USA) which measures accelerations [12].

The advantages of the use of these sensors in the prevention of damage that reduces the final quality of the products had led to the development of new equipment: the PTR-200, manufactured by SM Engineering, Denmark in 1999; the IRD 400 manufactured by Techmark, USA in 1999; and the Smart Spud, manufactured by Sensor Wireless (Canada) in 2000. All of them are equipped with tri-axial accelerometers [10].

IRD is mainly used in the fruit packing lines test [12]. Impact data must be related to bruise susceptibility of each fruit type by establishing impact damage thresholds of the products [13]. Garcia *et al.* [14,15] evaluated the effect of different materials and decelerator elements of the packing lines in fruit bruising, and obtained a reduction of 30 g in the mean impact. Van Canneyt *et al.* [16] found a model to relate the impact energy level of machinery (using a PRT-200) and potato tissue discolouration.

The IRD is frequently used in processing lines but has not been applied to evaluate harvest machinery. In the research reported in this article the IRD sensor (impact recorder device) was used to evaluate several tomato harvesters. Although some studies were made with these harvesters during the seventies in Spain, the present machines have improved technologically and have reduced considerably in size requiring further studies to characterise these new harvesters.

The specific objectives of this study were:

- to evaluate the impacts that the tomatoes suffer during mechanical harvest, using the IRD sensor;
- to determine the critical points at which damage occurs during harvesting;
- to assess the real damage produced by different harvester models.

## 2. Experimental Section

### 2.1. Tomato Fruit

There are two types of processing tomatoes: peeled and concentrated. Peeled tomatoes are elongated and canned whole, while concentrated tomatoes are spherical and canned triturated. Thus, peeled tomatoes need to be long shape varieties and high quality tomatoes with total absence of mechanical damages. On the other hand, concentrated tomatoes do not need to be specific shape varieties and slight mechanical damages are not so important, because damaged tomatoes can still be triturated and canned.

Tomato and IRD shape differences do not greatly influence the results of this research. Desmet *et al.* [17] used a spherical sensor PMS60, similar to IRD. The shape differences between the electronic fruit PMS60 and the tomatoes produced a very small overestimation of the prediction of the puncture injury due to the PMS60 behaviour (rolls freely). In this research, this overestimation was reduced because the quantity of tomato in each machine element during the harvest did not allow the IRD to roll. The radius of curvature influence the absorption of energy in an impact [18], but for high impacts (60 N, lower than recorded during the mechanical harvesting) the differences in the energy absorbed between spherical and elongated shape are only 5% higher in these ones [7].

In addition any overestimation would force adjustment of the machines to obtain better quality, undamaged tomatoes.

Several varieties of processing tomato were selected:

1. peeled tomato varieties: Oxford, Ercole, Hypeel, Nautilus and Soto;
2. concentrated tomato varieties: H9280, Bishop, H9144, Soprano, H9665, H9583, H9036, Malpica, Perfectpeel and Odin.

The tomatoes were grown in Navarre, Spain. Plants were transplanted in March and April, distributed in lines, using black mulching and drip irrigation. Harvest was carried out from August to October.

### 2.2. Tomato Harvesters

Five harvesters commonly used in Navarre were studied. For privacy reasons, these machines are only identified as A, B, C, D and E. The principal differences among these harvesters were in the shakers and in the position of the belts in the selection zones. There were also differences in the unloading unit, because each harvester model incorporated a different solution to avoid product loss during unloading. Table 1 shows the technical characteristics of each model studied.

**Figure 1.**
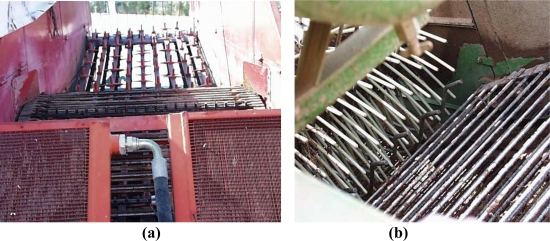
Harvester shaker systems: **(a)** belt shaker and **(b)** rotary shaker.

There are two types of shakers (Figure 1): those using belts or rotary shakers. Nowadays, there is a tendency to prefer rotary shakers instead of belt shakers, since they have a reduced size, require less energy in tomato selection and are more silent.

The belt shaker consists of several belts which produce horizontal and vertical vibrations in plants, allowing separation of the tomatoes. The gap between belts allows the fruits to fall to another belt to continue the selection process while the plant is expelled at the rear of the harvester.

The rotary shaker consists of a cylindrical rotor with gum rings located above. These rings contain a number of flexible equidistant sticks. During rotation, which is very slow, an engine moves two eccentric masses located at either side of the unit. They swing around two different and parallel axes, equidistant to the main rotation axis to which they are joined by a holder. The effect of these movements is to vibrate the plant.

As in the belt shaker, once the tomatoes are separated from the plant, they fall to a new belt. Plants are also transported by another belt to the rear of the harvester and expelled by means of powerful ventilators. The air flow also helps to eliminate leaves and parts of branches left in between tomatoes.

The ring number and the speed on the rotary shaker and vibration on the belt shaker can be modified for better adaptation of the harvest to crop conditions.

### 2.3. Impact Recording Device Description

**Figure 2.**
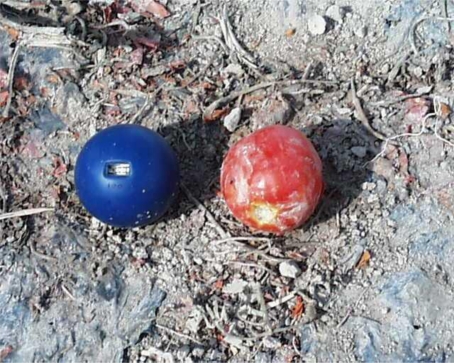
IRD sensor near a tomato fruit.

Impact measurement on tomatoes was done using the IRD-400 wireless sensor (Figure 2). It was developed at the Michigan Agricultural Experiment Station and the USDA’s Agricultural Research Service (United States). It is mainly used in fruit packing lines and consists of a tri-axial accelerometer as sensor, a microprocessor device designed for data collection, an internal real-time clock, and a rechargeable Ni-Cd battery [8].

The IRD-400 is a new version of the IS-100 and is manufactured by Techmark, Inc. It has a 3,906 Hz sample rate, a sensitivity of 6-300 g and an accuracy of 3%. A 64 mm diameter IRD was used because this size is the nearest to the size of commercial processing tomato fruits. The IRD-400 is controlled by the user using the PCIRD© software version 3.03, provided by Techmark, Inc. Using this software, the user defines the impact sensitivity and other data collection parameters, begins and stops impact recording, and uploads and interprets impact data [19]. The acceleration chosen to be considered zero impact was 50 g [20] because peaches and apples were not damaged by impacts under 50 g and 70 g, respectively, although the lowest impact intensity that can damage the tomatoes has not been established [21].

The IRD-400 records the following variables:

1. number of impacts (N);
2. a_max_ acceleration peak computed for each impact (data are reported relative to the acceleration due to gravity (g), where 1 g is equivalent to 9.80665 m/s^2^ [14]. Dividing the recorded acceleration by the standard acceleration of gravity (g in m/s^2^) the dimensionless variable a_max_ was obtained);
3. impact duration (duration);
4. instant at which the impact happens;
5. computed velocity change registered in each impact (Δv). It is the area under the acceleration-time curve, it is an absolute value, and it is expressed in m/s; and,
6. estimated direction of the impact, based on the orthogonal X, Y and Z axis.

The impact duration cannot be used to characterise impacts due to the ambiguity of impact completion [22]. Velocity change, the integration of the impact curve, tends to smooth the impact data and the error due to the lack of definition at the beginning and at the end of the impact curve. In this research, the angle of the impacts is not an important parameter because the tomatoes move free during harvesting and the angle of the impact changes from one to another tomato and in each harvester element. According to these authors, the variables maximum acceleration (intensity) and velocity change (a_max_ and Δv, respectively) are the best ones to explain or characterise the impacts.

The sphere’s kinetic energy changes during impact from its initial energy to zero, and then the energy is either absorbed by the impacting materials “inelastic impact” or is returned to the sphere “elastic impact” (or an intermediate “partially elastic impact”). In the first case the rebound velocity is 0 and in the second case the rebound velocity is equal to the impact velocity by in the opposite direction [23].

The variable Δv provides very important information to evaluate the risk of producing damage from a determinate impact against a determinate surface. When the IRD impacts at a determined velocity, V1, against an element of the combine the surface absorbs some of the impact energy if it is an absorbing surface, and then, the rebound velocity will be lower than V1 and it could even be zero. This latter case is an inelastic impact against a cushioning surface (e.g., rubbery surface). If the surface doesn’t absorb the energy (e.g., a steel surface) the rebound velocity will be similar to V1, and the velocity change (Δv) will be very small, the surface won’t absorb the impact and the damage risk increases.

### 2.4. Tests Methodology

The tests were carried out during four harvest periods in Navarre, Spain. In all tests, the following data relating to the machine and to the field were taken: location of the field; tomato variety and sanitary state; state of the field (stones, weeds…); harvester model, work velocity, and velocity of the belts in addition to the test data.

One test into an empty machine was performed each year for each harvester at the beginning of harvest including 40 IRD passages to characterize the impact in the harvester’s elements without tomatoes present. The working test included, at least, 40 IRD passages for each year and each machine during the harvest period, from August to October. Samples were taken from the field (control samples) and from each zone of the harvester.

Arazuri *et al.* [4] established the test method used to evaluate the machines: the IRD was attached to a plant, which was then collected by the cutting and lifting system and placed inside the harvester, so the IRD followed the whole route with the tomatoes and was dropped into the trailer.

During the process, all important times were recorded using a chronometer, which was started when the IRD went into the harvester and the times at which the IRD was in each one of the machine elements were recorded. Afterwards, comparison with the IRD registered times allowed us to determinate which the critical points were. Critical points are defined as the elements in which the IRD registered impacts higher than the limit explained above (50 g).

**Figure 3.**
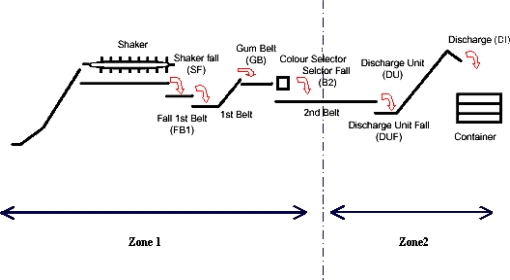
Harvester scheme: elements and zone distribution.

Although all elements or transfer points were tested (Figure 3) some of the main elements focused on were:

1. shaker: the tomatoes are separated from the plant by vibrations;
2. out of shaker: step between shaker and sorting belts;
3. sorting belts: rest of plants, stones, *etc.* were eliminated by hand;
4. drop to unloading unit: this unit received tomatoes from sorting and discharged them onto a trailer; and,
5. loading onto the trailer or discharge to transport.

The elements of the harvesters were divided in two general zones to evaluate the damages (Figure 3):

Zone 1: from the cutting system to the second belt.
Zone 2: from the second belt to loading onto the trailer.

Finally, tomato samples were taken during harvesting with two purposes:

1. to study the percentages of whole tomatoes (without damage), cracked tomatoes (damages in the skin) and broken tomatoes (deeper damages);
2. to analyse the influence of the harvester on the final quality of tomatoes. Samples were analysed using a Stable Microsystem TA-TX2 Texture Analyser (Stable Micro Systems Ltd., Surrey, UK) and an Impact Test System [24–26].

Size (diameters and length), colour (CieLab), acidity (ml citric acid/l) and soluble solids content (ºBrix) of the fruits were also measured.

For each test day and for each harvester, thirty fruit samples were taken for each treatment. The sample size is in accordance with ISO 874 and is sufficient for the estimated errors always to be lower than 10% [27].

The selected treatments were:

1. hand harvest sample: 1 m of a plant row was hand harvested (about three tomato plants). This sample was classified in four groups: red tomatoes, green tomatoes, over-ripened tomatoes and others and defined the damage level not produced by the harvesters.
2. the second belt: the sample was taken once the tomatoes had passed through the colour selection unit.
3. the upper zone or trailer: the sample was taken in the upper zone of the discharge box (container with a capacity of about 500 kg) or in the upper zone of the pool (about 5,000–6,000 kg containers).
4. the lower zone: the sample was taken in the lower zone of the container of discharge.

All samples taken from the harvester were classified into four groups:

1. whole tomatoes: fruits without mechanical damage;
2. cracked tomatoes: tomatoes with only skin damage
3. broken tomatoes: tomatoes with deeper damage (affecting the pulp);
4. others: with damage produced by insects, fungus, *etc.*

The statistical analysis was performed using the SPSS v.9 statistical program and the significance degree selected was p < 0.05. Significant differences between harvesters were determined using One-way ANOVA, and means comparison Duncan’s Test was used to identify similar groups between combine models. In addition, the coefficient of variance (the standard deviation divided by the mean) and the experimental error [27] were studied in order to check that the IRD’s data had enough accuracy. Finally, a multiple linear regression (MLR) was done to obtain damages prediction models from the IRD’s data. Although in this work only R^2^ is shown, other different parameters were used to evaluate the MLR models: prediction error, analysis of variance (p < 0.05) and residuals analysis.

In order to explain the obtained results as clearly as possible, the statistical study first determined where the impacts occurred in the combine harvester to define the critical points (Figure 3). Because of security, it was not possible to access to the different elements of the combine to take samples during the test, therefore the elements were grouped in two zones, as indicated above: zone 1 (from cutting system to second belt) and zone 2 (from second belt to loading onto the trailer).

The a_max_ quartiles were calculated and impact acceleration levels were classified, in order to identify small differences between harvesters, as:

1. low acceleration impacts: <67.9;
2. medium-low acceleration impacts: 67.9 to 89.1;
3. medium-high acceleration impacts: 89.1 to 130.6; and,
4. high acceleration impacts: >130.6.

## 3. Results and Discussion

The changes in tomato firmness and skin resistance produced by the harvester are due to the impacts against different parts of the machine. The level of the impact depends on both the type of the surface impacted and the impact energy. For a soft surface, the duration of the impact is long while the acceleration is low.

The coefficient of variation (the standard deviation divided by the mean) was between 50 and 60% due to the great impact variability inside the machine, the estimation error for the mean value of a_max_ and Δv was between 1.7 and 1.8% as a large number of impacts were observed. Among harvesters, E showed the highest estimation error, 8%, since in this case, the number of impact observed was lower. For the rest, the estimation errors varied between 2 and 5%.

Among harvester elements, the estimation errors increased as a consequence of a reduction in the number of impacts on each element. The error grew from 2 to 20% depending on the particular element.

### 3.1. Harvesting Test Using the IRD Sensor

**Figure 4.**
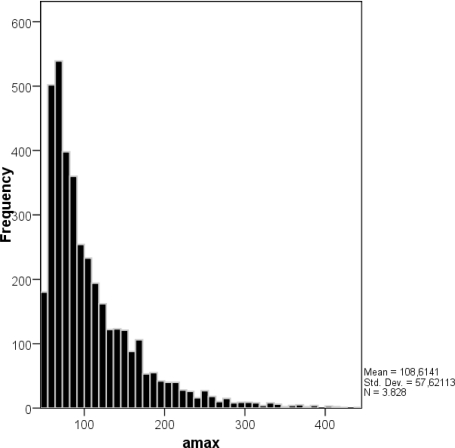
Distribution of the impacts according to the impact acceleration a_max_.

The majority of the registered impacts (Figure 4) had maximum accelerations values (a_max_) between 60 and 180. There were few impacts with a_max_ above 300, but there were some impacts that reached an a_max_ value of 440.

#### 3.1.1. Comparison of impacts according to the machine function and impact element

The highest impact accelerations were in the fall from the shaker (SF) (122.07) and the lowest impact accelerations occurred in the selection belts (Belt1, 76.84 and Belt2, 60.11).

The surfaces which better absorbed the impacts were in the zones receiving more intense impacts; e.g., the SF registered mean a_max_ value of 122.07 and mean Δv value of 1.76 m s-1 and in Belt2, a_max_ was 60.11 and Δv was 0.81 m s-1.

In the discharge onto the trailer (DI) impact levels at 95.30 for a_max_ were similar to those in other parts of the machine but the Δv value was the highest at 2.02 m s-1. The explanation is the tomato’s energy absorbing capability.

**Figure 5.**
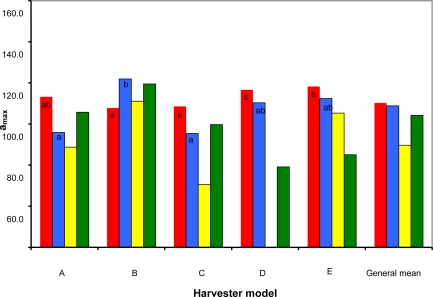
Impact acceleration (a_max_) for the harvester elements: 
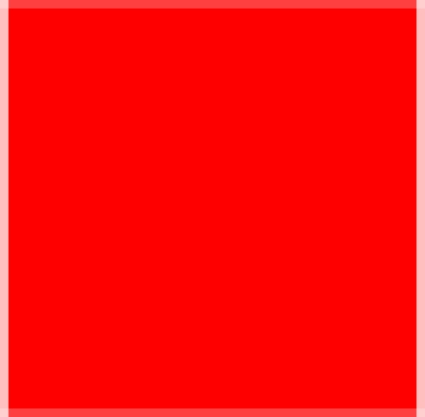
 shaker; 
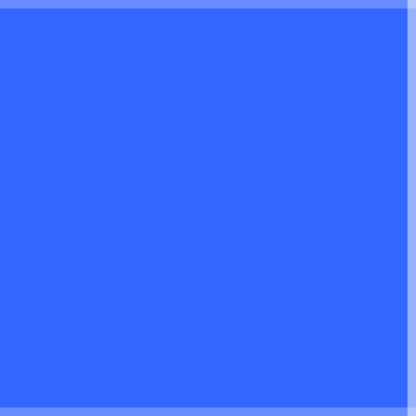
 discharge unit fall; 
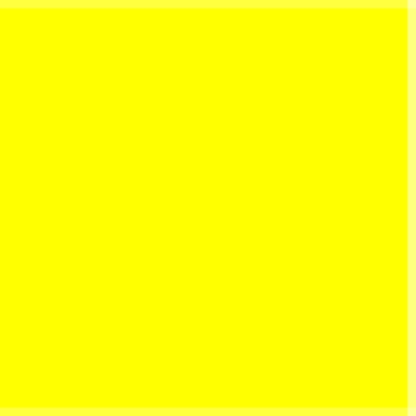
 discharge unit; 
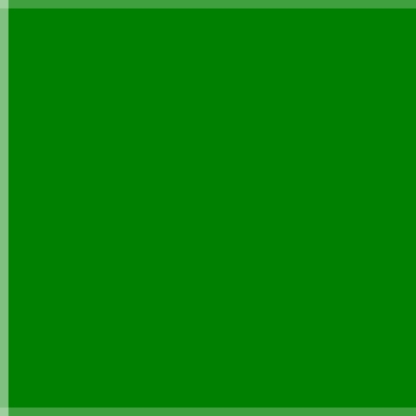
 discharge unit’s exit. Different letters in each column are significantly different (p < 0.05).

The differences in a_max_ between machines are shown in Figure 5:

1. Shaker: the average rates on this element could be classified as level 3 impacts, medium-high acceleration. Two groups were distinguished, E, D machines with a_max_ rates higher than 110 and, on the other hand, B and C with lower rates. The Δv variable (data not shown) also divided the machines into two groups. In the first one, were B and E (1.34 m s-1 and 1.38 m s-1, respectively), models in which the impacts happened on a less absorbing surface, –compared to greater than 1.6 m s-1 for the rest of the harvesters.
2. Discharge unit fall (DUF): in this transfer, although generally tomatoes fall on the harvested tomatoes, several a_max_ rates in B harvester exceeded 120. At this point, the highest risk of damage was found in the D harvester with an a_max_ value of 110.34 and a Δv value of 1.53 m s-1.
3. Discharge unit (DU): the registered impacts at this point would be the electronic fruit falls due to a bad position in the bucket belt, which is the discharge unit. The different harvesters showed similar rates of a_max_; but differences in Δv variable were found. In D harvester, no impacts have been registered while in C machine, DU was a risk element (70.56 and 0.71 m s-1 for a_max_ and Δv, respectively). Although in E the average a_max_ was 105, the Δv was significantly higher at 2.58 m s-1, and so the risk of damage was low.
4. The discharge unit’s exit (DUE): harvester models A and B showed the highest medium values for a_max_, 105.7 and 119,5, respectively, while the rest of combines showed lower medium values for this variable, 79.2 for D, 85.0 for E and 99.7 for C. On the other hand, A, C and D showed the lowest Δv values: 1,48 m s-1, 1.11 m s-1 and 1.38 m s-1, respectively. Therefore this element is a critical point for A combine because of the a_max_ high value and the low Δv value and also for the C combine because of the really low Δv value and the high a_max_ value.

#### 3.1.2. Comparison of impacts with regard to the machine and impact zone

**Figure 6.**
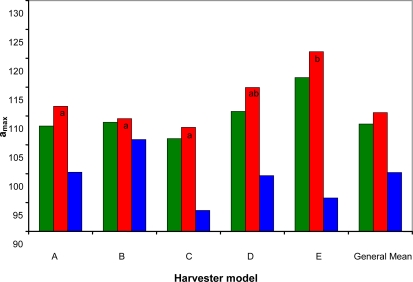
Impact acceleration (a_max_) for each harvester and each zone: 
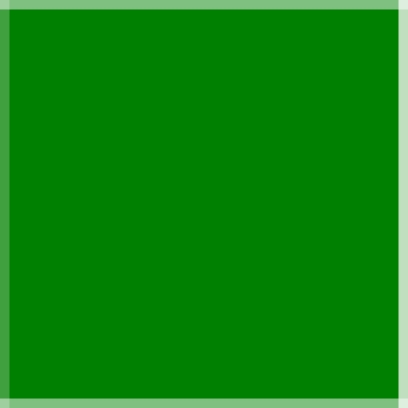
 harvester mean value; 
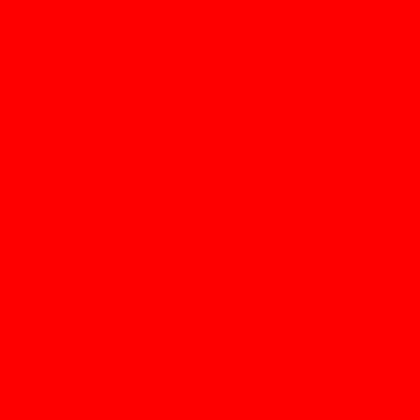
 zone 1; 
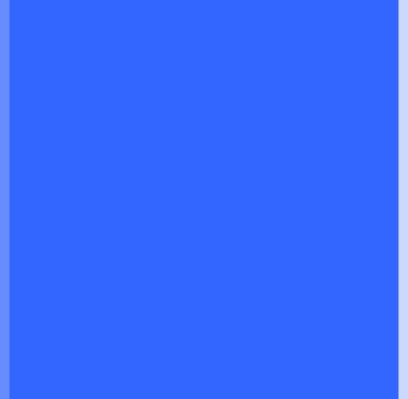
 zone 2. Zone 1: combine elements from cutting device to second belt. Zone 2: combine elements from second belt to loading onto the trailer. Different letters in each column are significantly different (p < 0.05).

**Figure 7.**
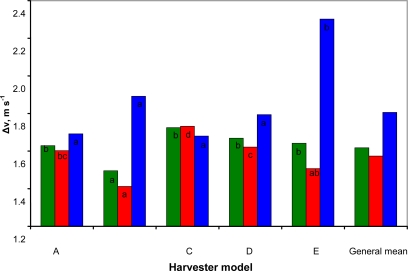
Velocity change (Δv, in m s^−1^) for each harvester and each zone: 
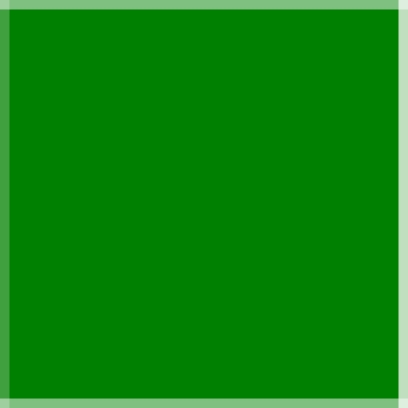
 harvester mean value; 
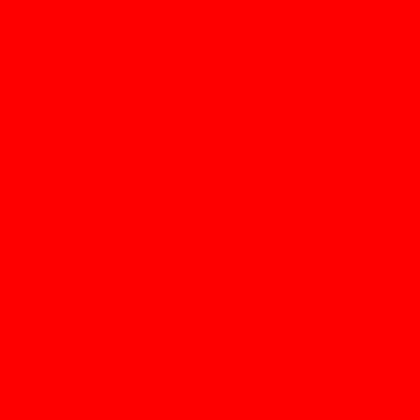
 zone 1; 
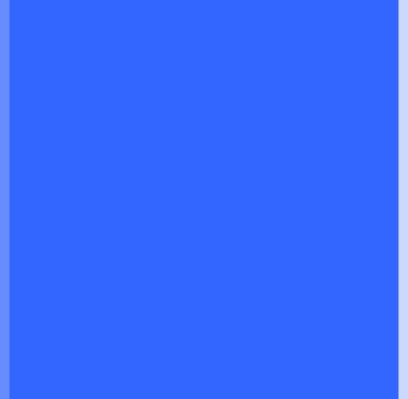
 zone 2. Zone 1: combine elements from cutting device to second belt. Zone 2: combine elements from second belt to loading onto the trailer. Different letters in each column are significantly different (p < 0.05).

The highest intensity impacts and the lowest velocity changes took place in zone 1, thus the risk of tomato damage was higher in this zone of the machines (Figure 6 and Figure 7). The colour selector in zone 1 was not used in these field assays because it eliminated the IRD as another strange element. That is why this step was done by hand, but in all cases the height and the conveyor belt material was the same. In order to solve this problem the selector was characterised in empty machine assays which revealed the colour selector as one of the most critical zones, causing an increase of differences between zones 1 and 2.

Figure 6 shows the impact intensity registered on each combine harvester and zone. Data distinguishes two groups in zone 1, with E having higher impact intensity compared to all other machines except D which is intermediate. In zone 2, there are no statistically significant differences between the five combine harvester models. There were no significant differences between the different combine models for harvester mean a_max_ variable.

The velocity change –Δv– showed more variation (Figure 7). In zone 1, the lower values for Δv were registered in B and E. Therefore, the impacts on less absorbing surfaces occurred in these harvesters. D and A showed medium values for this variable and C reached the highest values. In zone 2, E showed significantly higher Δv impacts.

Finally, Figure 6 shows the a_max_ data registered by the IRDs during the harvesting tests. The average impact acceleration values were between 106 and 116 for all the machines and the difference between models lies on the Δv variable (Figure 7). The harvester B, with a significantly lower value of Δv showed the greatest risk to damage tomatoes, because for the same intensity value, the impacting surface absorbed less energy.

#### 3.1.3. Proportion and number of impacts and a_max_ maximum values in each combine

The study of impacts at the different transition points and for the different machines was always done comparing the mean value obtained over the various test. Maximum values registered in each harvester and zones are showed in Table 2.

In zone 1, the highest maximum values for impact acceleration were registered (except for A), highlighting the 440.7 value in E. The lowest maximum value was 365.5 in D. In zone 2, generally the maximum a_max_ values were lower, though in A it exceeded 400. The lowest value was registered in E at 179.1.

Zone 1 is the most aggressive from the impact intensity point of view and the one where the impact number is the highest. Moreover, it is a critical zone because of the greater proportion of impacts occurring there (Table 2). The B, C and E machines exceeded 80% impact proportion in this zone.

The medium-high intense impacts were predominant in B and C. And finally, the proportion of high impacts (>130.63 a_max_ value) was higher in E and D than others, which leads to an increase of damage risk in these machines.

For the different elements of the machine (not shown in the table) the shaker and the shaker fall (SF) registered the highest a_max_ maximum in almost all machines (about 407 and 332). Other parts, such as the DUF (discharge unit fall) and discharge (DI), also exceeded 300 a_max_ value. With regard to discharge, the machine is not as important as its control. It would be the driver’s responsibility to unload at a suitable height.

The shaker registered the highest maximum and the proportion of impacts occurring there exceeded 50% in all cases.

### 3.2. Study of the Total Number of Damages Observed in Tomatoes

#### 3.2.1. Damages produced in each combine

**Figure 8.**
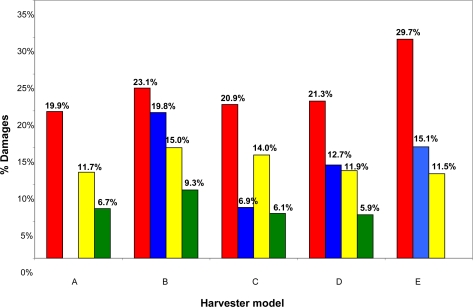
Total damages (%) recorded for each harvester and each year: 
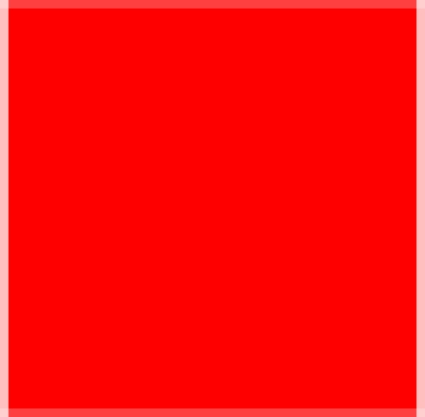
 year 1; 
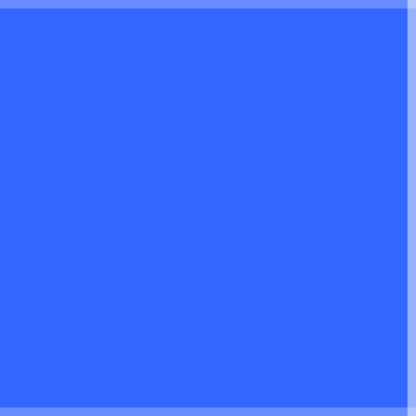
 year 2; 
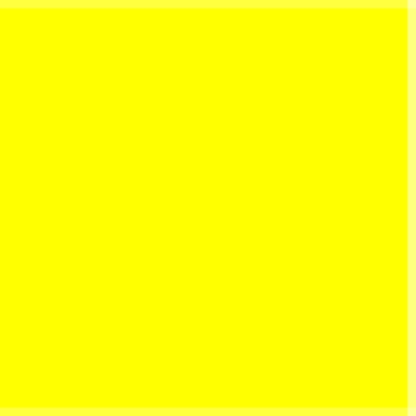
 year 3; 
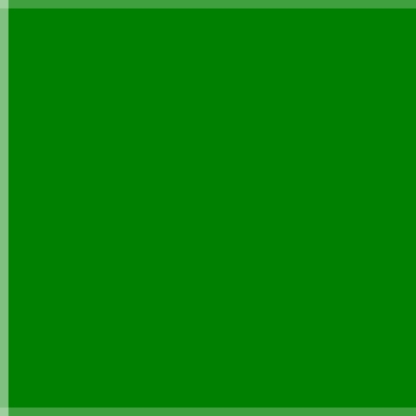
 year 4.

The total percentage of damages/year produced in each harvester did not exceed 30% (Figure 8). With the exception of C, that produced very low damage in the second year, the tendency was for damage to decrease year by year. In the second year, damages data were not recorded for harvester A.

From the results of this research, the critical points for each harvester model were identified and it was possible to provide the drivers the specific indications for each harvester model. For the harvester models E and D, the IRD identified the zone 1 as the critical zone, being the shaker the critical point, therefore, the velocity of the shaker was reduced. For the harvester model B the IRD identified risk of damage in the discharge unit and in the discharge unit exit, therefore, a rubbery implement was installed to reduce the discharge height and the discharge velocity and the advance speed was reduced. For the harvester models A and C, the IRD identified the discharge unit’s exit as the critical point because of the high impact medium acceleration for A and the low velocity change for C, therefore, a rubbery implement was installed to reduce the discharge height and the discharge velocity. By means of these specific regulations, in the last four years, the percentage of damaged tomatoes was reduced from 20 to 29% to less than 10%. Particularly, the damages were reduced to 6.7% for combine A in the last year and to 6.1% for C.

**Figure 9.**
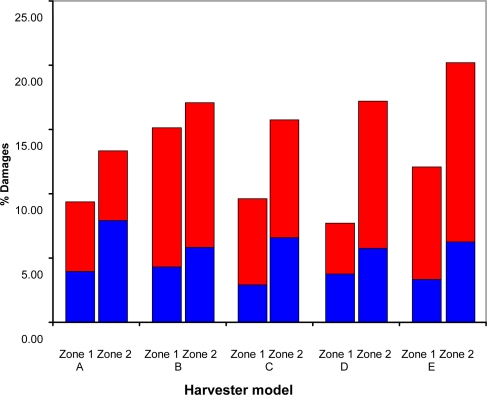
Total damages registered per harvester and zone: 
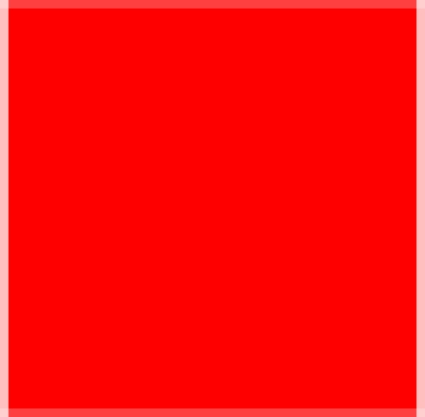
 % broken; 
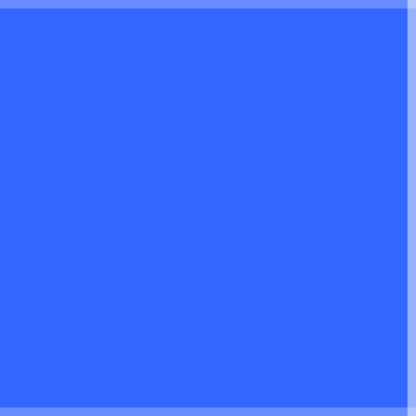
 % cracked. Zone 1: combine elements from cutting device to second belt. Zone 2: combine elements from second belt to loading onto the trailer. Cracked tomatoes with only skin damages. Broken tomatoes with deep damages.

The identification of the zones where damages occur is very important. According to the obtained data using the IRDs, in zone 1 the highest intensity impacts happened. The effects are shown in Figure 9. The percentage of cracked tomatoes was similar in all machines, but there were differences concerning the broken tomatoes percentage. B showed the highest percentage of broken tomatoes in zone 1 and D showed the lowest rate. However, these differences were not significant.

Figure 9 shows the percentages of cracked and broken tomatoes in each harvester during the discharge (zone 2). These data do not include the cracked and broken tomatoes from zone 1, which were previously eliminated (the total number of tomatoes damaged in zone 2 were corrected using the percentage of tomatoes damaged in zone 1). Thus, it is assured that the damages observed in the trailer were produced in zone 2 of the harvester. The lowest percentage of cracked tomatoes was found in D and the highest in A. With regard to broken tomatoes the highest rate was in E and the lowest in A. However, the data didn’t show significant differences among harvesters.

#### 3.2.2. Relation of the obtained data by IRD, laboratory results and field damages

As a result of the laboratory and field tests, prediction models of the percentage of damaged tomatoes and the IRD evaluation of the machines were established. These models (Equation 1 and equation 2) showed coefficients of determination (R^2^) from 0.91 to 0.99. They allow estimation of percentages of cracked and damage tomatoes basing on IRD sensor and laboratory data, harvester and tomato information, respectively. Therefore the percentage of damages at harvest time would be previously known:

(1) $$\%\;\text{cracked}=-162.5+0.1\;{\text{a}}_{\text{max}}-6.7\;\Delta \text{v}+0.15\;\text{TL}+6.3\;\text{RI}+41.8\;\text{FN}$$

where:

% cracked = percentage of cracked tomatoes (%)
a_max_ = acceleration peak (dimensionless)
Δv = velocity change (m s-1)
TL = tomato length (mm)
RI = ripeness index, related to the tomato acidity (ml citric acid/l)
FN = firmness, related to the variables from the non-destructive compression test carried out with the Texture Analyser, (N)

(2) $$\%\;\text{damages}\;=\;-108.5+0.4\;{\text{a}}_{\text{max}}+53.5\;\Delta \text{v}+2\;\text{RI}-3.9\;\text{FN}$$

where:

% damages = physical damages observed in tomatoes.

A summary of these data is shown in Table 3.

This is highly important for the canning industry that manufactures in Navarre (Spain) whole tomatoes, a good product that yields a better price in the market. From the analysis of the percentages of damaged tomatoes and the a_max_ values from each test it is possible to check that the a_max_ statistical mode increased when the percentage of damage increased from values about 40 for percentages of damage below 10% to values about 60 for percentages of damage over 20%. Thus, the most frequent impacts did not damage the tomatoes. Therefore the impacts happened in the critical points are the one that determinated the percentage of damage which makes very important the study of the specific critical points that has been developed in the reported research. The mean a_max_ values increased when the percentage of damage increased. The mean a_max_ values were 100.6, 110.5 and 115.4 for percentages of damage below 10%, between 10% and 20% and over 20%, respectively. Therefore it could be possible to establish a threshold for a_max_ above which the percentage of damage probably will be too high and could affect to the final tomato quality.

## 4. Conclusions

The IRD sensor, in spite of being designed to evaluate handling lines, can be used to evaluate tomato harvesters. The coefficient of variation for variables a_max_ and Δv exceeded 50%. However, the sample size allows maximum errors for estimation of 11% with regards to the average of the variable. In the working tests, the error varied from 2% to 8%, depending on the model of the combine harvester. It has been possible to identify the critical points, where the damages were produced, for each one of the five harvester models. The highest risk of damage was in zone 1 of the combine because the impacts were of higher intensity and hit less absorbing surfaces than in zone 2. The shaker and exit from the shaker are two of the harvester elements that registered the highest intensity impacts. The highest intensity impacts hit the most absorbing surfaces, diminishing the risk of damage inside the harvesters. By adjusting, in a specific way each harvester model, using the results from this research, it has been possible to reduce the tomato damage percentage from 20 to 29% to less than 10%.

## Figures and Tables

**Table 1.** Technical characteristics of the studied tomato harvesters.

*Characteristics*	*A*	*B*	*C*	*D*	*E*
Maker	Diesel	John Deere Diesel	Iveco-Aifo-Diesel	Fiat-Iveco-Diesel	Fiat-Diesel
Power, kW	110.4	93	88.32	88.32	88.32
Working speed, km h^−1^	10	7.5	8	9	6
Transfers speed, km h^−1^	20	30	25	24	22
Brakes	Servo-brakes	Disc on oil bath	Hydraulic on oil bath	Hydraulics	Hydraulics
Transmission	Hydrostatic	Hydrostatic	Hydrostatic	Hydrostatic	Hydrostatic
Steering radius, m	---	4	3.7	4.5	---
Dimensions, m	9.8 × 3.18 × 3.25	10.2 × 2.8 × 3.4	9.5 × 2.5 × 3.3	9 × 2.5 × 3.3	9.45 × 2.49 × 3.5
Pitch, m	2.75	3	2.5	2.7	2.75
Track, m	1.5	1.65	1.5	1.5	1.57
Weight, kg	9,000	8,800	8,000	7,800	8,320
Production capacity, t ha^−1^	35	20	25	30	---
Max unloading height, m	3.4	3.25	3.3	3.6	3.2
Shaking system	Belts	Rotating roller	Rotating roller	Belts	Rotating roller

**Table 2.** Maximum impact acceleration data, proportion of impacts and number of impacts by zone and level for each combine.

		Zone 1(a)	Zone 2	<67.9(b)	67.9–89.1	89.1–130.6	>130.6
A	Maximum a_max_(c)	404.3	408.5	67.8	89.1	129.0	408.5
	Proportion, %(d)	70.1	29.9	31.5	24.0	21.2	23.2
	Number impacts(e)	4791	2044	2153	1640	1449	1593
B	Maximum a_max_	405.6	333.7	67.9	89.0	130.6	405.6
	Proportion, %	82.6	17.4	23.3	26.3	25.9	24.4
	Number impacts	5994	1263	1691	1909	1880	1778
C	Maximum a_max_	421.0	336.3	67.8	89.1	130.6	421.0
	Proportion, %	86.4	13.6	24.4	23.7	26.8	25.1
	Number impacts	6181	973	1746	1695	1917	1796
D	Maximum a_max_	365.5	310.6	67.8	88.8	129.4	365.5
	Proportion., %	72.9	27.1	25.1	24.8	23.8	26.3
	Number impacts	5046	1876	1737	1717	1647	1820
E	Maximum a_max_	440.7	179.1	67.7	88.6	129.9	440.7
	Proportion., %	82.3	17.7	24.1	26.3	21.1	28. 5
	Number impacts	4917	1058	1440	1571	1261	1703

(a) Zone 1: combine elements from cutting device to second belt. Zone 2: combine elements from second belt to loading onto the trailer

(b) Impact level.

(c) Maximum value of the acceleration recorded in each impact (a_max_).

(d) Proportion of impacts in each zone and level, in %.

(e) Number of impacts registered in each zone and level.

**Table 3.** Summary of laboratory data (mean and standard deviation): Tomato length (mm), firmness (N) and ripeness index (mL citric acid/L), for each harvester model.

Harvester model	Tomato length (mm)	Ripeness index (mL citric acid/L)	Firmness (N)
A	58.4 ± 10.7	3.15 ± 1.0	11.8 ± 3.8
B	62.8 ± 11.6	3.07 ± 0.8	9.56 ± 3.6
C	61.5 ± 9.2	3.4 ± 0.7	9.9 ± 3.3
D	58.7 ± 8.7	3.6 ± 0.7	9.9 ± 3.2
E	53.9 ± 10.8	3.5 ± 0.9	11.2 ± 3.4
